# RIPK1 or RIPK3 deletion prevents progressive neuronal cell death and improves memory function after traumatic brain injury

**DOI:** 10.1186/s40478-021-01236-0

**Published:** 2021-08-17

**Authors:** Antonia Clarissa Wehn, Igor Khalin, Marco Duering, Farida Hellal, Carsten Culmsee, Peter Vandenabeele, Nikolaus Plesnila, Nicole Angela Terpolilli

**Affiliations:** 1 Institute for Stroke and Dementia Research (ISD), LMU Klinikum, Ludwig-Maximilians University Munich, Feodor-Lynen-Str. 17, 81377 Munich, Germany; 2 Munich Cluster of Systems Neurology (SyNergy), Munich, Germany; 3 Institute for Pharmacology and Clinical Pharmacy, Biochemical-Pharmacological Center Marburg, University of Marburg, Karl-von-Frisch Straße 2 K03, 35032 Marburg, Germany; 4 University of Marburg, Marburg, Germany; 5 Molecular Signaling and Cell Death Unit, VIB-UGent Center for Inflammation Research, UGent-VIB Research Building FSVM, Technologiepark 71, 9052 Ghent, Belgium; 6 Department of Biomedical Molecular Biology, Ghent University, Ghent, Belgium; 7 Graduate School of Systemic Neurosciences (GSN), Ludwig-Maximilians University Munich, Munich, Germany; 8 Department of Neurosurgery, LMU Klinikum, Ludwig-Maximilians University Munich, Munich, Germany; 9 Present Address: Medical Image Analysis Center (MIAC AG) and Qbig, Department of Biomedical Engineering, University of Basel, Basel, Switzerland

**Keywords:** Traumatic brain injury, Chronic posttraumatic brain damage, Magnetic resonance imaging, Necroptosis, Ferroptosis, Neuroprotection

## Abstract

Traumatic brain injury (TBI) causes acute and subacute tissue damage, but is also associated with chronic inflammation and progressive loss of brain tissue months and years after the initial event. The trigger and the subsequent molecular mechanisms causing chronic brain injury after TBI are not well understood. The aim of the current study was therefore to investigate the hypothesis that necroptosis, a form a programmed cell death mediated by the interaction of Receptor Interacting Protein Kinases (RIPK) 1 and 3, is involved in this process. Neuron-specific RIPK1- or RIPK3-deficient mice and their wild-type littermates were subjected to experimental TBI by controlled cortical impact. Posttraumatic brain damage and functional outcome were assessed longitudinally by repetitive magnetic resonance imaging (MRI) and behavioral tests (beam walk, Barnes maze, and tail suspension), respectively, for up to three months after injury. Thereafter, brains were investigated by immunohistochemistry for the necroptotic marker phosphorylated mixed lineage kinase like protein(pMLKL) and activation of astrocytes and microglia. WT mice showed progressive chronic brain damage in cortex and hippocampus and increased levels of pMLKL after TBI. Chronic brain damage occurred almost exclusively in areas with iron deposits and was significantly reduced in RIPK1- or RIPK3-deficient mice by up to 80%. Neuroprotection was accompanied by a reduction of astrocyte and microglia activation and improved memory function. The data of the current study suggest that progressive chronic brain damage and cognitive decline after TBI depend on the expression of RIPK1/3 in neurons. Hence, inhibition of necroptosis signaling may represent a novel therapeutic target for the prevention of chronic post-traumatic brain damage.

**Supplementary Information:**

The online version contains supplementary material available at 10.1186/s40478-021-01236-0.

## Introduction

With an estimated case load of 69 million per year [1], traumatic brain injury (TBI) represents a leading cause of death and disability in all age groups worldwide, especially in children and young adults. The incidence of TBI is expected to increase in the coming decades, as the number of the two main etiologies—motor vehicle accidents and falls—are expected to rise due to an increase in motorization and an aging population, respectively [2]. The socio-economic impact of TBI is vast, with estimated costs of approximately 400 billion US$ annually [3]. This number does not only include the direct costs due to acute primary care, but also long-term follow-up costs since many TBI survivors suffer from mood changes, memory deficits, and loss of fine motor skills, have difficulties returning to their previous occupation, and therefore require lifelong support [4–6]. Consequently, TBI is increasingly recognized as a chronic neurological disorder with socio-economic implications comparable to conditions like Alzheimer’s disease or other neurodegenerative disorders [7].

While the pathophysiology of acute brain damage has been investigated in detail in experimental animals and in humans over the past decades, relatively little is known about the mechanisms determining long-term outcome after TBI. Chronic functional deficits in TBI patients may be caused by progressive brain atrophy in cortex and hippocampus [8, 9] and hydrocephalus formation [10–13]. So far, clinical and experimental studies suggest that inflammation plays an important role for the development of chronic posttraumatic brain damage [14–18], however, the cellular and molecular mechanisms downstream of this process are not fully understood. Particularly, the trigger and the intracellular signaling cascades causing neuronal cell death weeks and months after TBI are still unknown.

Necroptosis is a form of necrotic regulated cell death, which involves the upstream assembly of the necroptosome complex formed by the interaction of receptor interacting protein kinase 1 and 3 (RIPK1 and 3) [19] and downstream RIPK3-mediated phosphorylation of mixed lineage kinase like protein (MLKL). Necroptosis can be initiated by activation of Toll-like receptors (TLR) 3 and 4, TNF-alpha receptor 1 or, as more recently shown by us and others, by cylindromatosis (CYLD)-mediated deubiquitination of RIPK1 [20]. CYLD is prone to activation by reactive oxygen species (ROS) [21]. Hence, necroptosis may be activated by several events, TNF release, TLR activation, inflammation and ROS production, which are all believed to occur in the brain following TBI [16, 22, 23]. Based on these findings, we hypothesize, that necroptosis may be a relevant intracellular mechanism which triggers chronic neurodegeneration after TBI. To address this issue, we used mice deficient for RIPK1 in neurons or RIPK3 and investigated lesion progression by longitudinal magnetic resonance imaging (MRI), behavioral outcome, and necroptotic signaling up to three months after TBI in a clinically relevant mouse model of TBI. Our results demonstrate that necroptosis is an important novel mediator of chronic neurodegeneration after TBI.

## Material and methods

## Ethical statement

All animal experiments were reviewed and approved by the Ethical Review Board of the Government of Upper Bavaria. The results of the study are reported in accordance with the ARRIVE guidelines [24]. Animal husbandry, health screens, and hygiene management checks were performed in accordance with Federation of European Laboratory Animal Science Associations (FELASA) guidelines and recommendations [25]. Only male mice between 6 and 8 weeks old mice were used. All surgical procedures, behavioral testing, imaging, and data analysis were performed in a randomized fashion by a researcher blinded to the genotype and group allocation of the animals. Group allocation was obtained by drawing lots by a third party not involved in the study or data analysis.

### Animals

Inducible neuronal *Ripk1* cKO mice were generated as previously described [26] and bred as *Ripk1*fl/fl (WT) and *Ripk1*fl/fl::Camk2a-Cre + /Cre (cKO) in our facility. For induction of neuronal RIPK1 deletion, *Ripk1* cKO mice (starting at 4 weeks of age) received three intraperitoneal injections of 2 mg Tamoxifen (Sigma-Aldrich, Taufkirchen, Germany, # T5648) in a 100 µl Miglyol suspension (Caelo, Hilden, Germany, #3274) every 48 h (day 1, 3, 5). RIPK3-deficient mice were generated and kindly provided by V. M. Dixit, Genentech Inc., San Francisco, CA, [27] and bred heterozygously to obtain *Ripk3*^−/−^ (KO) and *Ripk3*^+/+^ (WT) cohorts. The genotype of each RIPK1 and RIPK3 deficient mouse was proven by genotyping (Additional file 1: Figure S1a and b). PCR was performed using the AccuStartTM II Mouse Genotyping Kit (Quanta Biosciences, Beverly, MA, #95,135–500) according to the manufacturer’s instructions. Primers were obtained by Metabion (metabion GmbH Planegg, Germany). Neuronal specific conditional knock-out of *Ripk1* was further proven by immunohistochemistry for RIPK1 (Additional file 1: Figure S1c). Induction of neuronal specific Cre recombinase resulted in an 80% reduction of RIPK1 expression in cortical neurons (Additional file 1: Figure S1d). Neither neuronal RIPK1 nor global RIPK3 deficient mice had any obvious phenotype and were born at normal Mendelian distributions.

### Controlled Cortical Impact model of traumatic brain injury

Animals were subjected to experimental traumatic brain injury using the previously described Controlled Cortical Impact (CCI) method [23, 28–30]. CCI induces a highly reproducible focal lesion and causes progressive brain damage and cognitive decline thereby replicating many acute and chronic characteristics of human TBI [23]. In short, after induction of anesthesia with buprenorphine (0.1 mg/kg Bw) and isoflurane (4%, 30 s), animals were sedated with 1.5–2.5% isoflurane in 30% oxygen and 70% nitrogen under continuous monitoring of body temperature and heart rate. After right parietal craniotomy, the impact was directly applied to the intact dura with a pressure-driven steel piston with a diameter of 3 mm (L. Kopacz, University of Mainz, Germany; 8 m/s impact velocity, 1 mm penetration depth, 150 ms contact time). For sham-surgery, the piston was placed on the dura, but no impact was applied. The craniotomy was resealed with tissue glue (VetBond, 3 M animal care products, St. Paul, MN) and animals were kept in an incubator at 34 °C and 60% air humidity until they regained full motor activity in order to prevent hypothermia. Carprofen (4 mg/kg every 24 h) was administered i.p. for the following 72 h for analgesia.

### Experimental time-line

**Fig. 1:**
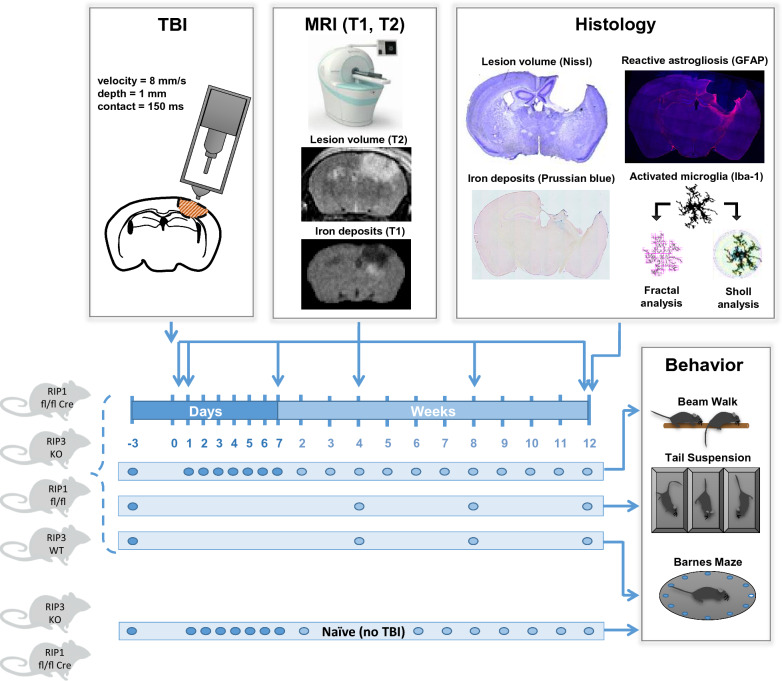
Experimental groups, methods, and time line

Motor function, depression-like behavior, and memory function were evaluated three days before trauma to obtain baseline values and up to three months thereafter. Lesion volume and tissue iron was evaluated by repetitive MRI up to three months after injury and validated by histology. At the end of the observation time brains were removed for immunohistochemical analysis of lesion volume, tissue iron, astrocyte activation, and microglia morphology (Fig. 1).

### Body weight and general condition

Animals were weighed daily from three days before CCI until day 7 after trauma, then weekly. General condition, surgery wounds, behavior, nutrition, and fluid balance were checked daily in the early postoperative period, then weekly.

### Magnetic resonance imaging and analysis

For longitudinal determination of lesion volume, MRI measurements were performed 15 min, 24 h, 7 days, one, two, and three months after TBI. For all animals, T1 weighted, T2 weighted, and diffusion weighted imaging (DWI) sequences were collected as previously described [23] under isoflurane anesthesia (1–1.5% in 30% oxygen/70% nitrogen) and multimodal monitoring of physiological parameters using a 3 T nanoScan® PET/MR (Mediso, Münster Germany). Sequences were collected in the following order: T2-weighted imaging (2D fast-spin echo (FSE), TR/TE = 3000/57.1 ms, averages 14, matrix size = 96 × 96; field of view = 16 mm × 16 mm; slice thickness = 500 µm, interslice gap = 60 µm), T1-weighted imaging (2D fast-spin echo (FSE), TR/TE = 610/28,6 ms, averages 14, matrix size = 96 × 96; field of view = 16 mm × 16 mm; slice thickness = 500 µm, interslice gap = 60 µm). Total imaging time was approximately 35 min per mouse and time-point. Lesion volume was measured using ImageJ software (Rasband, W.S., ImageJ, U. S. National Institutes of Health, Bethesda, Maryland, USA, https://imagej.nih.gov/ij/, 1997–2018) in T2 sequences. 14 slices surrounding the lesion were chosen for each dataset and the area segmented using the polygon tool. Volume was then calculated using the following equation:

$$V \, = \, d*\left( {A1/2 \, + \, A2 \, + \, A3 \ldots + \, An/2} \right)$$

with d being the distance between slices in mm (slice thickness + interslice gap), and A being the measured area in mm^3^.

Hippocampal atrophy was assessed in T2-weighted images as previously described [23]. One section located in the center of the lesion containing the hippocampus was chosen at the same position for each animal. Areas of both hippocampi were segmented using the polygon tool and the ipsilesional area of the hippocampus was expressed as % of the area of the uninjured contralateral hippocampus.

### Behavioral testing

To exclude age-related factors as a cause for behavioral changes during the observation period, all behavioral tests were performed with an additional control group of non-traumatized (naive) RIPK3 or RIPK1 deficient mice (Fig. 1).

### Motor function—Beam Walk

The Beam Walk Test was performed as previously described [23, 28, 30] on a 1 m long and 1 cm wide suspended wooden rod. Time to cross the beam and the number of missteps was recorded. Animals with more than two missteps in pre-trauma testing were excluded from randomization.

### Memory and learning behavior—Barnes Maze

The Barnes Maze test, a well-established paradigm for assessing memory function [31, 32], was performed 1, 2, and 3 months after CCI as previously described [23]. In short, the animal was placed on a brightly lit round platform with 20 identical holes along its outer rim and trained to locate a box affixed below one of the apertures (home cage) as fast as possible. Time to reach the home cage (latency) as well as distance travelled and walking speed were recorded and analyzed using a video tracking software (EthoVison XT©, Version 11, 2014 Noldus Information Technology). Animals were trained for four consecutive days and memory function was evaluated on the sixth day.

### Tail Suspension test

The Tail Suspension test is a paradigm to assess depression-like behavior in rodents and was performed as previously described [23]. Briefly, animals were suspended by the tail for three minutes and their movements recorded and analyzed using a video tracking software (EthoVison XT©, Version 11, 2014, Noldus Information Technology). The time of inactivity was used as a proxy for depression-like behavior [33].

### Histological assessment

#### Lesion volume/ hippocampus volume

Three months after TBI, animals were fixed with 4% PFA in deep anesthesia by transcardial perfusion. Fourteen sequential 50 µm thick coronal sections were cut at 500 µm intervals on a vibratome (Leica, Germany) in order to match the tissue volume investigated by MRI, stained with cresyl violet according to Nissl, and evaluated by histomorphometry for lesion volume using the following formula as previously described [23]:

$$V \, = \, d*\left( {A1/2 \, + \, A2 \, + \, A3 \ldots + \, An/2} \right)$$

The volume of the hippocampus in the traumatized hemisphere was determined in six slices one mm anterior until four mm posterior to bregma and then normalized to the contralesional side.

### Iron deposits

One section per animal was stained with Prussian blue (Iron Stain Kit, Sigma-Aldrich, # HT20) at -1.5 mm from bregma to visualize iron deposits using a light microscope (Axioscope, Carl Zeiss Microscopy GmbH, Jena Germany). Tile scans were then processed in ImageJ by using the color deconvolution tool to separate color channels. The blue channel was binarized and the integrated density was measured using the particle analysis plugin. Values of the traumatized hemisphere were expressed as percentage of the contralateral side.

### Immunohistochemistry

Fifty micrometer thick floating coronal sections were prepared as previously described [34]. Blocking and incubation with the primary antibody was performed in 1% bovine serum albumin, 0.1% gelatin from cold water fish skin, 0.5% Triton X-100 in 0.01 M PBS at pH 7.2–7.4 for 72 h at 4 °C. The following primary antibodies were used: IBA-1 (rabbit, Wako, #019–19,741, 1:200), GFAP-Cy3 (mouse, Sigma Aldrich, #2905, 1:200), RIPK1 (rabbit, Novusbio, #NBP1-77077SS, 1:100), NeuN (guinea pig, Synaptic Systems, #266 004), and phosphorylated MLKL (rabbit, Cell signaling technology, # 91689S, 1:100). After incubation, sections were washed in PBS and incubated with the following secondary antibodies: anti-rabbit coupled to Alexa-fluor 594 (goat anti-rabbit, Thermo Fisher Scientific, #A-11012), anti-guinea pig coupled to Alexa-fluor 488 (goat anti-guinea pig, Thermo Fisher Scientific, # A-11073), and anti-mouse coupled to Alexa-fluor 647 (goat anti-mouse, Thermo Fisher Scientific, #A- 32,728) in 0.01 M PBS at pH 7.2–7.4 containing 0.05% Tween 20. Nuclei were stained with 4’,6-Diamidin-2-phenylindol (DAPI, Invitrogen, #D1306) 1:10,000 in 0.01 M PBS.

Imaging was performed using a ZEISS LSM 900 confocal microscope (Carl Zeiss Microscopy GmbH, Jena Germany). GFAP staining was recorded using a 10 × objective (EC Plan-Neofluar 10x/0.30 Pol M27) with an image matrix of 512 × 512 pixel, a pixel scaling of 0.2 × 0.2 μm and a depth of 8 bit. Whole brain images were collected in z-stacks as tile scans with a slice-distance of 2 μm and a total range of 14 μm [35]. For microglia analysis, images were acquired using a 40 × objective (EC Plan-Neofluar 40x/1.30 Oil DIC M27) with an image matrix of 1024 × 1024 pixel, a pixel scaling of 0.2 × 0.2 μm and a depth of 8 bit. Specific regions of interest were collected in Z-stacks to include the entire slice thickness with a slice-distance of 0.4 μm at 100 μm away from the lesion in the hippocampus and at 300 μm in the cortex. For p-MLKL staining, a 5 × objective was used (EC Plan-Neofluar 5x/0.16 Pol M27) with an image matrix of 1434 × 1434 pixel, a pixel scaling of 3.321 × 3.321 μm and a depth of 8 bit. Whole brain images were collected in z-stacks as tile scans with a slice-distance of 5 μm and a total range of 25 μm. To demonstrate the intracellular localization of p-MLKL, a 100 × objective (Epiplan-Neofluar 100x/1.3 Oil Pol M27) was used with an image matrix of 512 × 512 pixel, a pixel scaling of 0.166 × 0.166 μm and a depth of 8 bit. Images were collected in z-stacks with a slice-distance of 0,280 μm and a total range of 5.6 μm. After obtaining a maximum intensity projection, images were imported into ImageJ [36] and intensity of p-MLKL measured in the rim of the lesion and normalized to the signal of DAPI to correct for differences in staining. The corrected signal was then normalized to the same sized region of interest on the contralateral hemisphere.

### Analysis of astrocyte coverage

Assessment of astrocyte coverage was performed using ImageJ in sections stained for GFAP (see above). Z -stacks were imported into Fiji and split into individual channels. GFAP intensity in five ROI (250 × 250 μm; at 0, 250, 500, 750, and 1000 μm distance from the lesion in the striatum) was then measured using the mean grey value and normalized to the measurements of the contralesional hemisphere to adjust for possible differences in staining intensity.

### Analysis of microglia coverage and morphology

Microglia coverage was manually assessed in maximum intensity projections of iba-1 stained sections. One section per animal was chosen at 1.5 mm from bregma and two ROIs chosen on the ipsilesional hemisphere, one in layer V of the cortex at 300 µm away from the lesion and one in the CA1a region of the hippocampus. The number of microglia was normalized to total DAPI positive cell count and expressed in as percentage of coverage in sham operated animals.

To assess microglia morphology, Sholl and fractal analysis were performed to indicate ramification, cell range, total cell size, and circularity using a modified protocol from Young and Morrison [37]. Z-stack images were converted to a maximum intensity projection and cells were individually cut out using the polygon selection tool in ImageJ [35]. Only cells fully captured within the z-stack were selected. After background subtraction, images were binarized and resized to 600 × 600 pixels keeping the original scale. Speckles or debris around the cells were removed using the paintbrush tool. Sholl analysis was performed using the Sholl analysis plugin in ImageJ [38]. Centered on the soma, concentric circles with an increasing radius of 2 μm were drawn, the number of intersections measured at each radius. After converting binary images to outlines, fractal analysis was performed using the FracLac plugin for ImageJ [39]. As described previously [37], the total number of pixels present in the cell image of either the filled or outlined binary image were calculated and later transformed to μm^2^ (pixel area = 0.208 μm^2^). Cell circularity was calculated as Circularity = 4*π*Area/Perimeter^2^. Maximum span across the convex hull represents the maximum distance between two points in the convex hull.

### Statistical analysis

Sample size was calculated with the following parameters: alpha error = 0.05, beta error = 0.2, calculated standard deviation ranged from 15 to 20% (depending on the parameter investigated), and biologically relevant difference = 30%. All data is given as mean ± standard deviation (SD) if not indicated otherwise. For comparison between groups, Student t-test was used for normally distributed data and Mann–Whitney Rank Sum test for non-normally distributed data according to the result of Shapiro–Wilk normality test. Measurements over time were tested between groups using One-way or Two-way ANOVA for Repeated Measurements, followed by Tukey’s multiple comparisons test for normally and Holm-Sidak's multiple comparisons test for non-normally distributed data as post hoc test. Calculations were performed with Sigma Plot version 14.0 (Systat Software GmbH, Erkrath, Germany).

## Results

A total of 33 male RIP1 deficient mice (naïve RIP1^fl/fl Cre^ group: *n* = 4, RIP1^fl/fl Cre^ and RIP1^fl/fl^ sham groups = 5 each, CCI RIP1^fl/fl^ group: *n* = 10, RIP1^fl/fl^ Cre group: *n* = 9) and 32 male RIP3 deficient mice (naïve RIP3^−/−^, RIP3^+/+^, and RIP3^−/−^ sham group: *n* = 5 each, CCI RIP3^+/+^ group n = 9, CCI RIP3^−/−^ group *n* = 8) were operated, assessed, and analyzed for the present study. One RIP3^+/+^ animal was excluded from randomization and not used for the study due to its performance in the Beam Walk Test before TBI (more than 2 missteps at baseline).

### Traumatic brain injury induces long-term necroptotic signaling in neurons

**Fig. 2:**
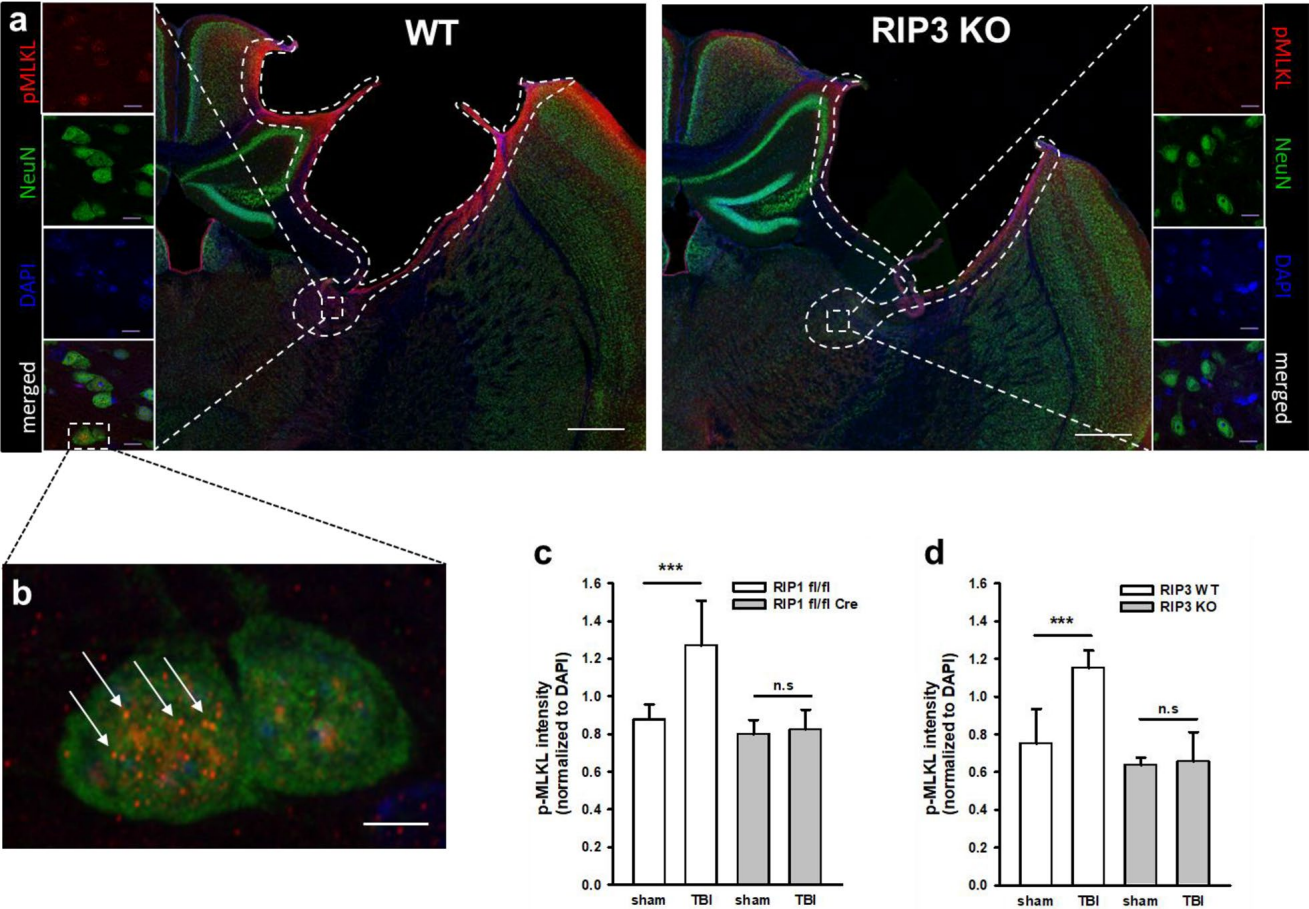
Phosphorylated MLKL, a marker of necroptosis is reduced in RIP knockout animals. **a.** Exemplary staining of pMLKL and NeuN (upper panel) and a pMLKL positive neuron at higher magnification (lower panel) in the rim of a traumatic contusion in a wild type mouse. **b.** pMLKL is significantly reduced in a RIPK3 deficient mouse. **c** and **d** p-MLKL signal intensity was significantly increased after TBI in wild type animals (white bars) compared to sham animals indicating significant presence of necroptosis three months after TBI; this increase was significantly blunted in RIPK1 (**c**) as well as in RIPK3 **d** mice where there was no difference between TBI and sham animals. Data are presented as mean ± SD; n = 9–10 for RIPK1, n = 8–9 for RIPK3. Two-way RM ANOVA with Tukey’s multiple comparisons test was used. ***p < 0.001, n.s. indicates no significant statistical difference between groups

Phosphorylated MLKL (pMLKL) was used as a specific marker for necroptosis [40]. Three months after TBI large amounts of pMLKL were found in the rim of the traumatic cavity, the presumed site of progressive chronic post-trauma brain damage (Fig. 2a, upper panel). pMLKL was found by high resolution confocal imaging in the cytoplasm of selected neurons as small dots, suggesting that pMLKL is part of a protein complex such as the necrosome (Fig. 2a, lower panel, white arrowheads). pMLKL staining was almost absent in neuronal RIPK3 deficient mice suggesting that necroptotic signaling in neurons did essentially not occur in these animals (Fig. 2b). Quantification of pMLKL staining showed a highly significant increase of activated MLKL in wild type mice of both strains, while neuronal *Ripk1* or global *Ripk3* knock-out completely blunted this response (Fig. 2c and d).

### Chronic posttraumatic brain damage is reduced in RIPK1 or RIPK3 deficient mice

**Fig. 3:**
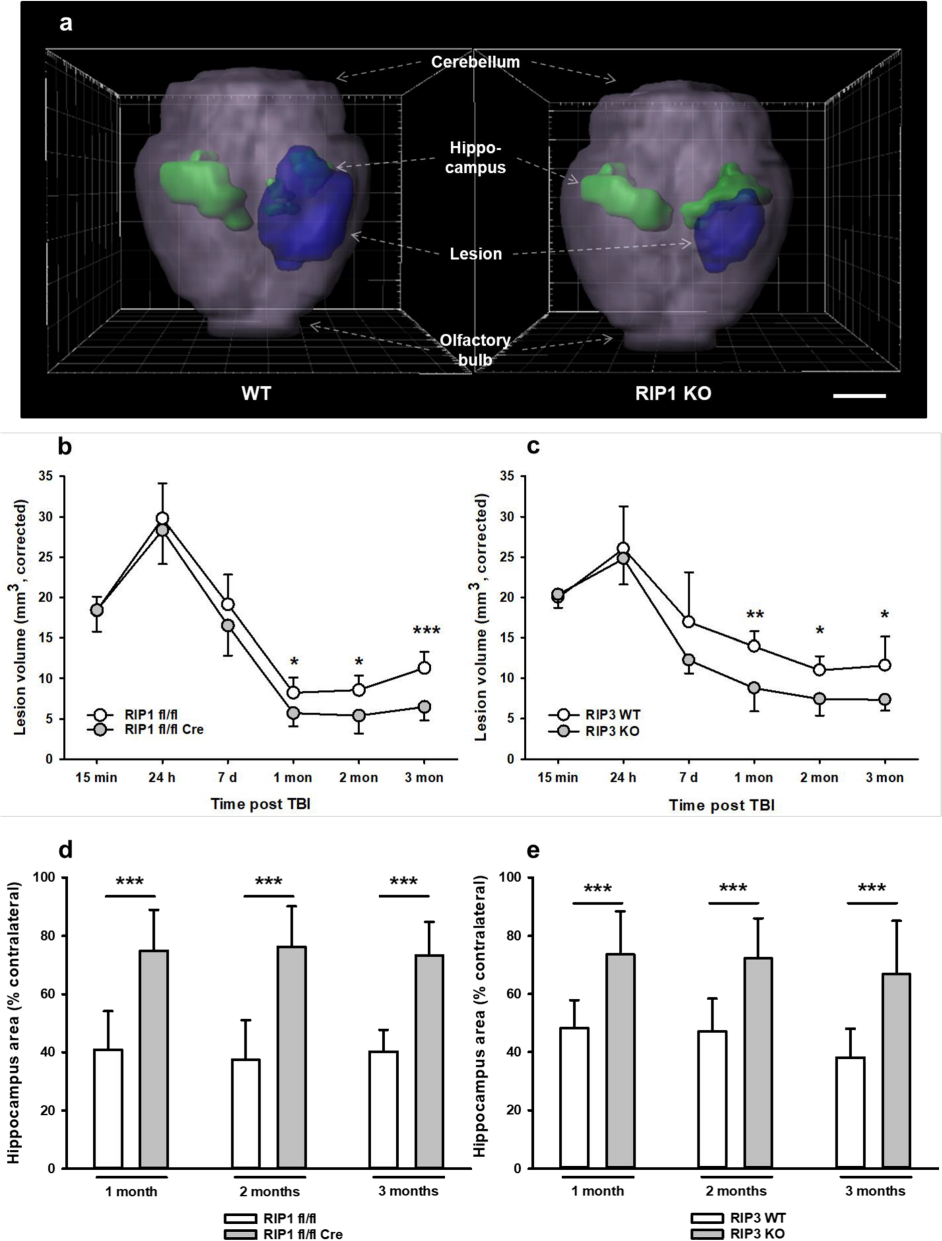
RIPK1 and RIPK3 deficiency significantly reduces posttraumatic brain damage. **a** 3D reconstruction of lesion volume (blue) in relation to ipsi- and contralateral hippocampus (green) for a RIPK3 wild type and a RIPK3 deficient mouse 3 months after TBI. Scale bar = 5 mm. **b** and **c** Lesion volume over time quantified by repetitive T2-weighted MR imaging in RIPK1 (**b**) and RIPK3 (**c**) knockout animal. **d** and **e.** Hippocampal atrophy over time assessed in longitudinal T2-weighted MRI. RIPK1 (**d**) and RIPK3 (**e**) knockout mice show better preservation of hippocampal tissue over time. Mean ± SD; n = 9–10 for RIPK1, n = 8–9 for RIPK3. Two-way RM ANOVA with Sidak's multiple comparisons test was used. *p < 0.05, **p < 0.005, ***p < 0.001

After demonstrating neuronal necroptotic signaling three months after TBI in wild type mice and showing that RIPK1 or 3 deficiency prevented this process, we evaluated lesion volume in cortex and hippocampus of wild type and RIPK deficient mice by longitudinal MR imaging. Two animals (one *Ripk1* fl/fl::CamK2a Cre and one *Ripk3* WT) of the study cohort died for unknown reasons before TBI and were excluded from analysis. All other animals completed the study. Anesthesia and craniotomy did not have any influence on general outcome parameters: sham-operated and CCI animals recovered equally well from surgery in terms of bodyweight (*Ripk1* cKO: Additional file 1: Figure S2a, *Ripk3* KO: Additional file 1: Figure S2b) and general health score (*Ripk1* cKO: Additional file 1: Figure S2c, *Ripk3* KO: Additional file 1: Figure S2d). Exemplary three dimensional reconstructions of the brain showed a large lesion and a small hippocampus in the ipsilateral hemisphere of traumatized wild type mice three months after TBI, while the lesion was significantly smaller and the hippocampus significantly larger in neuronal RIPK1 deficient mice (Fig. 3a). Longitudinal investigation of lesion size and hippocampal volume by repetitive MRI showed that the primary damage measured 15 min after trauma was comparable in RIPK1 deficient mice and their respective wild type controls (Fig. 3b, t = 15 min: *Ripk1* cKO: 18.4 ± 2.6 mm^3^, wt: 18.4 ± 1.7 mm^3^) indicating that the initial trauma was similar in all investigated animals. In agreement with previous results in this model, lesion size peaked 24 h after TBI in both experimental groups (*Ripk1* cKO: 27.0 ± 1.8 mm^3^, + 47% vs. 15 min; wt: 29.8 ± 4.3 mm^3^, + 61% vs. 15 min) as a representation of acute secondary brain damage. Lesion volume was not different between wild type and neuronal RIPK1 deficient mice at this time point indicating that necroptosis does not play a significant role for acute lesion progression. Within the first month after TBI, removal of necrotic tissue and scar formation resulted in an apparent shrinkage of the lesion. One to two months after TBI progressive loss of brain tissue started to occur, a process we previously demonstrated to continue for at least one year after experimental trauma [23]. Chronic post-trauma tissue injury was significantly reduced in neuronal RIPK1 deficient mice as compared to their wild type littermate controls (*t* = 1 month: *Ripk1* cKO: 5.7 ± 1.6 mm^3^, wt 8.2 ± 1.9 mm^3^, *p* = 0.0355, *t* = 3 mon *Ripk1* cKO: 6.5 ± 1.7 mm^3^, wt: 11.3 ± 2.0 mm^3^, *p* = 0.0002). A similar dynamic was seen in RIPK3 deficient animals. Acute injury was not affected by RIP 3 knock-out (Fig. 3c  *t* = 24 h, *Ripk3* KO: 24,8 ± 3,3 mm^3^, wt: 26,1 ± 5,3 mm^3^), while chronic lesion progression was significantly attenuated from one to three months after TBI (*t* = 1 month: *Ripk3* KO: 8.8 ± 2.9 mm^3^, wt: 14.0 ± 1.9 mm^3^, *p* = 0.006; *t* = 3 months: *Ripk3* KO: 7.3 ± 1.3 mm^3^, wt: 11.6 ± 3.6 mm^3^, *p* = 0.04). Individual traces for lesion volumes in each animal are given in Additional file 1: Figure S3a (RIP1 KO) and Additional file 1: Figure S3b (RIP3 KO). The MRI findings were corroborated by histopathological evaluations, i.e. acute brain damage 24 h after TBI was similar in RIP deficient and wild type animals (Additional file 1: Figure S4), while chronic brain damage three month after TBI correlated well with the injury assessed by MRI (Additional file 1: Figure S5a and b) and was significantly reduced in both knock-out strains (Additional file 1: Figure S5c and d).

Since memory deficits are a hallmark of chronic posttraumatic brain damage in mice and TBI patients [23, 41, 42], we next investigated long-term hippocampal damage by MRI. In the current TBI model, the hippocampus is only marginally injured acutely after TBI, but severely affected by progressive chronic damage as previously shown [23]. All wild type mice showed significant loss of hippocampal tissue in the traumatized hemisphere already one month after TBI (Fig. 3d and e, open bars). Starting one month after trauma, hippocampal loss was significantly less pronounced in neuronal RIPK1 and global RIPK3 deficient mice (Fig. 3d, RIPK1: reduction to 74.9 ± 18.5%, wt: reduction to 40.3 ± 18.7% of contralateral hippocampus, *p* = 0.0004; Fig. 3e, RIPK3: reduction to 78.0 ± 30.7%, wt: reduction to 48.2% ± 19.6% of contralateral hippocampus, *p* = 0.01). This significant difference persisted until the end of the observation period three months after TBI and was corroborated by histology (Additional file 1: Figure S5e and f).

### Astrogliosis and microglial activation are reduced in RIPK1 and RIPK3 deficient mice

**Fig. 4:**
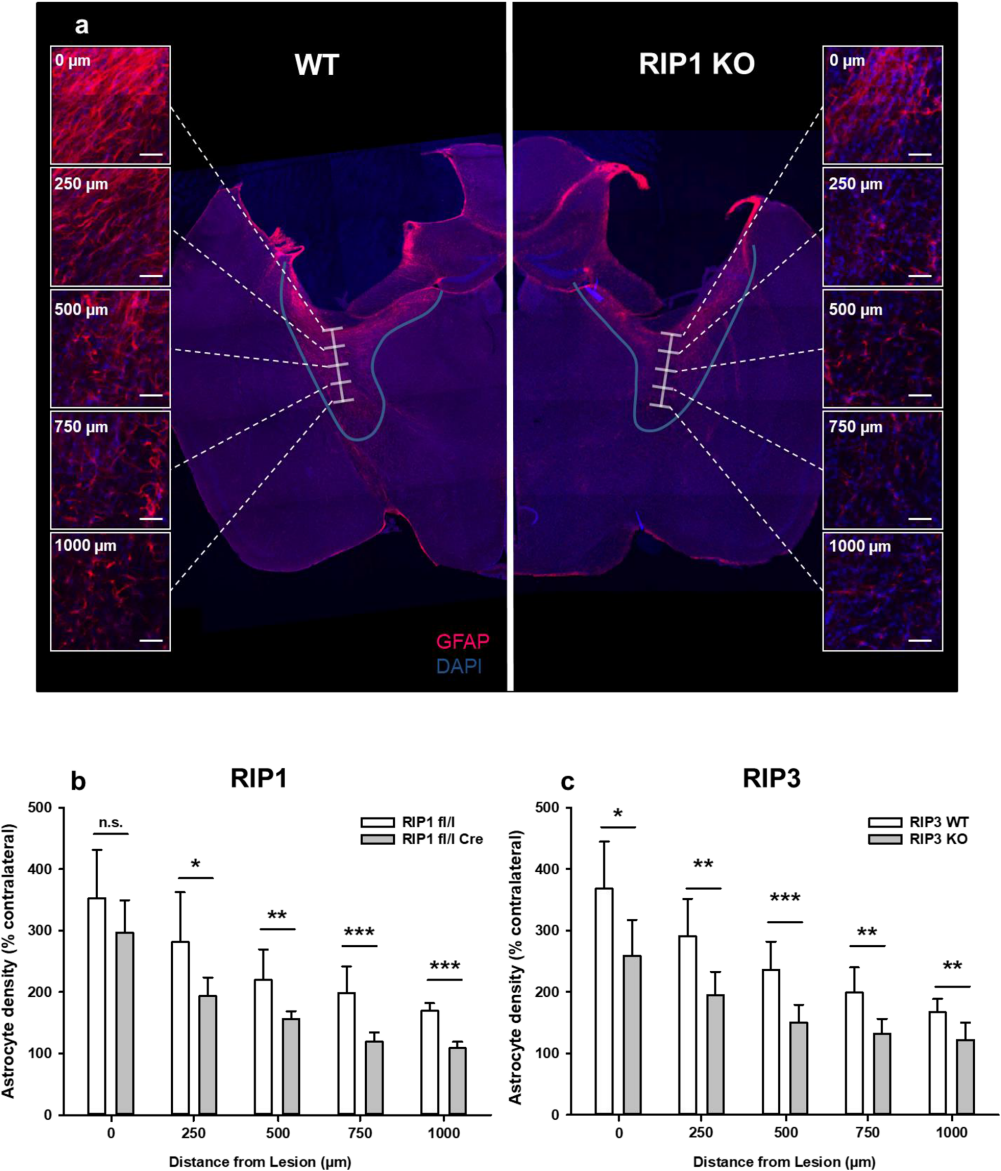
RIPK1 and RIPK3 deficiency reduces reactive astrogliosis three months after TBI. **a** Exemplary GFAP stainings in a wild type (left) and a RIPK1 knockout (right) animal at different distances from the traumatic contusion. Both images show the right hemisphere, but the left image has been mirrored along the midline for better visualization. Scale bar = 20 µm. Significant astrogliosis is present in the WT mouse, while it is heavily reduced in the neuronal RIPK1 deficient mouse. **b** and **c** Quantification of astrocyte coverage at different distances from the lesion. Astrocyte coverage was decreased in neuronal RIPK1 (**b**) and global RIPK3 (**c**) knockout animals compared to controls. Data are presented as mean ± SD; n = 9–10 for RIPK1, n = 8–9 for RIPK3. Students t-test for parametric and Man-Whitney-Rank-Sum test for non-parametric data were used. *p < 0.05, **p < 0.005, ***p < 0.001

The formation of a glial scar and the activation of microglia are other hallmarks of chronic brain damage after TBI [43, 44]. Indeed, we observed a marked increase in glial fibrillary acid protein (GFAP), an astrocyte marker, in the rim of the traumatic cavity and in perilesional tissue in wild type mice three months after TBI (Fig. 4a, left panel). Quantification of GFAP expression showed an almost four-fold increase in the rim of the lesion with a decreasing intensity towards perilesional areas (Fig. 4b and c, open bars). GFAP expression was far less pronounced in neuronal specific RIPK1 deficient mice (Fig. 4a, right panel). Quantification of astrocyte density by pixel-based analysis corroborated these findings and reveled that activation of astrocytes was significantly decreased by 25–35% in neuronal specific RIPK1 and in global RIPK3 deficient mice (Fig. 4b and c, closed bars).

**Fig. 5:**
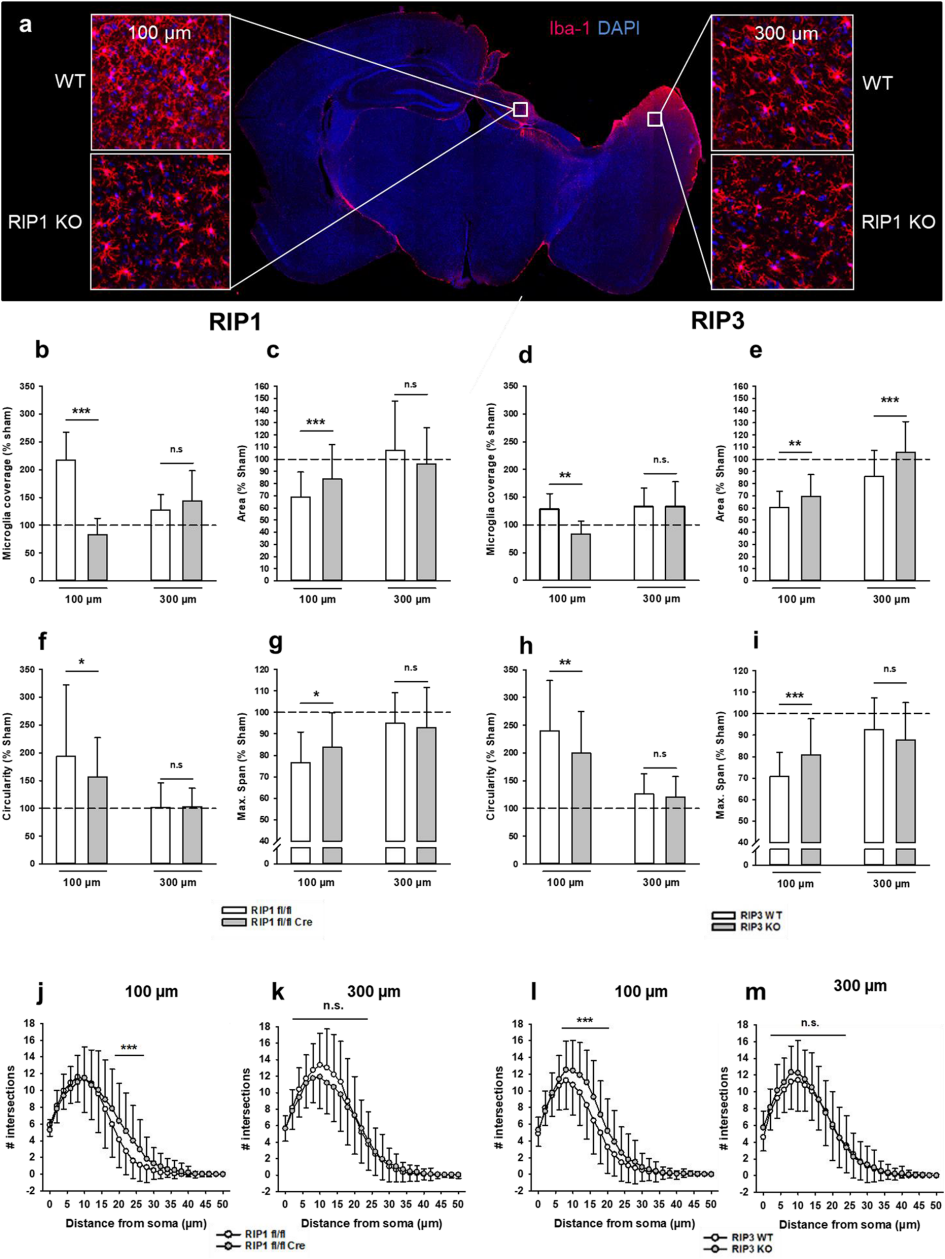
RIPK1 and RIPK3 deficiency reduces microglia activation. **a.** Exemplary stainings for the microglia marker iba1 in WT (upper inserts) and neuronal RIPK1 deficient mice (lower inserts) 100 µm (left inserts) and 300 µm (right inserts) from the rim of the lesion. **b-i.** Coverage and fractal analysis of microglia. In areas closer to the lesion site (100 µm, left side of each panel), knockout animals of both lines showed a decrease in microglia coverage (**b.** RIP 1, **d.** RIPK3) compared to their wild type littermates. Fractal analysis revealed that microglia of knockout animals in proximity to the lesion have less processes (**c.** RIP 1**, e.** RIPK3) are less circular **(f.** RIP 1, **h.** RIPK3), and overall smaller (**g.** RIP 1**, i.** RIPK3). In the more distal region, cells resembled those in sham operated animals, with no differences between genotypes. **j-m.** Sholl analysis also shows increased ramification, i. e. more active cells, close to the lesion (**j.** RIP 1**, l.** RIPK3), but not further away from the lesion site (**k.** RIP 1**, m.** RIPK3). Data are presented as mean ± SD; n = 9–10 for RIPK1, n = 8–9 for RIPK3. Student t-test for normalized and Man-Whitney-Rank-Sum-test for non-normalized data was used. *p < 0.05, **p < 0.005, ***p < 0.001. n.s. indicates no significant statistical difference between groups

Staining for the microglia marker Ionized calcium-binding adaptor molecule 1 yielded similar but more lesion associated findings three months after TBI (Iba1; Fig. 5). In wild type mice Iba-1 staining was most pronounced within a distance of 100 µm from the rim of the traumatic cavity, while only subtle changes were observed in areas 300 µm away from the lesion site (Fig. 5, WT). The density of iba-1 staining was heavily reduced in neuronal specific RIPK1 deficient mice (Fig. 5, *Ripk1* cKO). To quantify these changes, we assessed tissue coverage, area, circularity, and maximal span of microglia in the rim (100 µm) and in the vicinity (300 µm) of the traumatic lesion (Fig. 5b–i). In wild type mice all investigated parameters pointed towards a significant activation of microglia near the rim of the lesion, i.e. the coverage and circularity increased, while the area and the maximal span of microglia decreased (Fig. 5b–i, open bars). Microglia activation and the number of microglial branch points were significantly reduced and partly normalized in neuronal specific RIPK1 and in global RIPK3 deficient mice (Fig. 5b–m, closed symbols), suggesting that less neuronal cell death was associated with less microglial activation.

### Neuronal RIPK1 and RIPK3 deficiency improves cognitive outcome three months after TBI

**Fig. 6:**
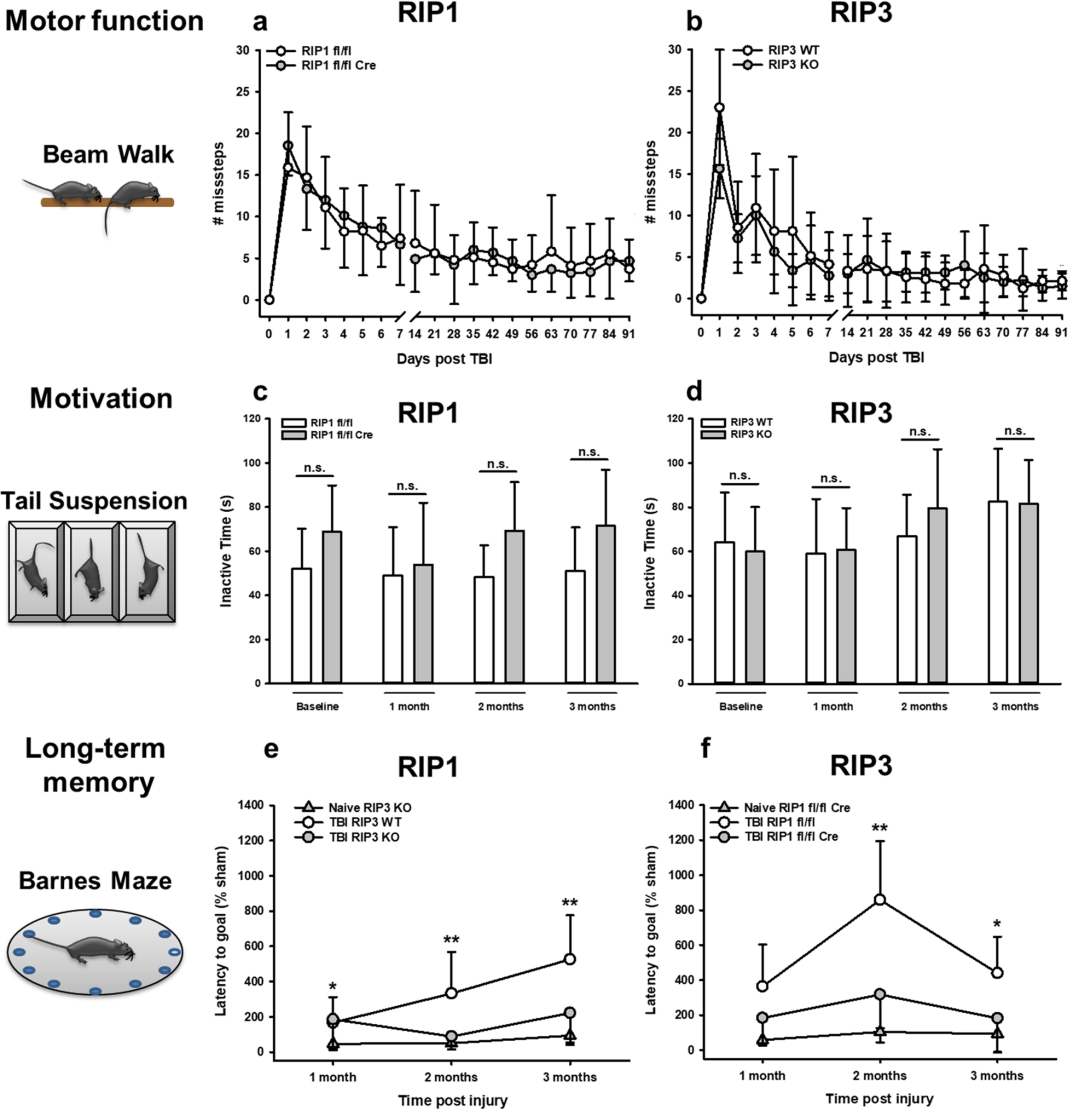
RIPK1 and RIPK3 deficiency improves neurocognitive performance three months after TBI. **a** and **b** Motor impairment after TBI. Beam Walk Test revealed long term impaired motor function of the left hind limb in TBI animals (missteps compared to respective baseline, # for WT, * for KO), but there were no differences between **a** RIPK1 or **b** RIPK3 knockout animals and their respective controls. **c** and **d** Depression-like behavior after TBI. Mice of both strains showed an increase of total immobility time throughout the time course of three months, however no significant differences could be detected between RIPK1 (**c**) and RIPK3 (**d**) knockout animals compared to wild type. **e** and **f** Learning and memory dysfunction after TBI. CCI induces severe long-term memory deficits in WT mice (open circles) while RIPK1 or RIPK3 knockout animals (grey circles) show similar long-term memory function as uninjured littermate controls. Data are presented as mean ± SD; n = 9–10 for RIPK1, n = 8–9 for RIPK3. Two-way RM ANOVA with Tukey’s multiple comparisons test was used. *p < 0.05, **p < 0.005, ***p < 0.001, ****p < 0.0001

To investigate whether the reduction of lesion size, hippocampal damage, scar formation, and microglial activation had an effect of functional outcome, we investigated motor function by beam walk, depression-like behavior by the Tail Suspension test, and long-term memory using the Barnes Maze test. TBI significantly deteriorated motor function and induced depression-like behavior compared to pretrauma performance as previously described [23], genetic deletion of RIPK1 or RIPK3, however, had no effect on these parameters (Fig. 6a–d). Long-term memory was normal in non-traumatized *Ripk1* or *Ripk3* knock-out mice; it was, however, significantly disturbed in traumatized animals, i.e. TBI increased the time needed to find the home cage, the latency to goal, by more than ten times and memory loss progressed over time (Fig. 6e and f, triangles and open circles). In neuronal specific RIPK1 and in global RIPK3 deficient mice, however, long-term memory function was almost completely preserved and resembled that of not traumatized animals (Fig. 6e and f, closed circles). Hence, protection of hippocampal neurons by genetic deletion of RIP 1 or RIPK3 resulted in preserved long-term memory function.

### Chronic lesion progression after TBI is associated with iron deposits and reduced in RIP-deficient mice

**Fig. 7:**
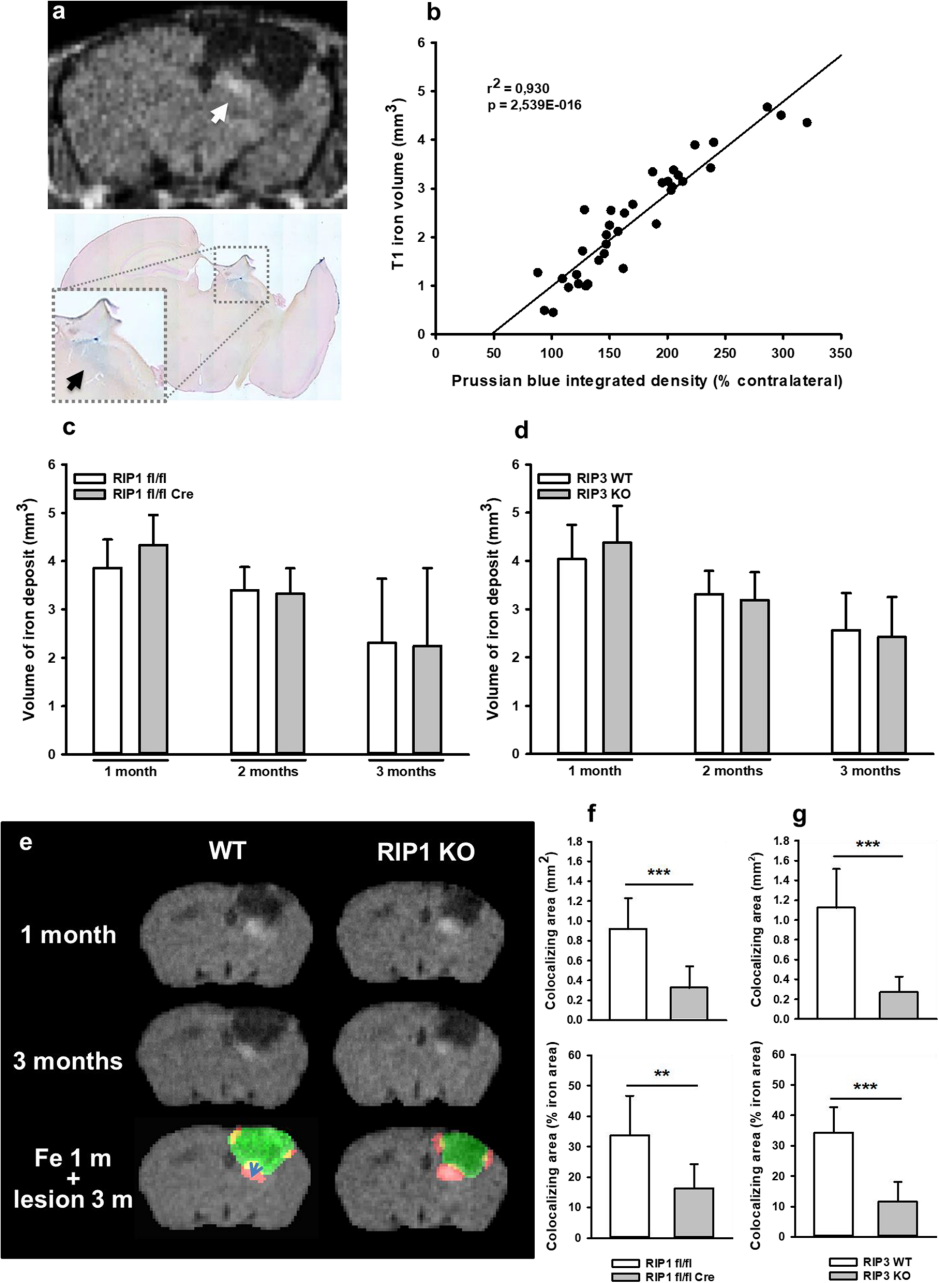
Lesion progression occurs in areas with iron deposits. **a** T1-weighted MRI (upper panel) and Prussian blue staining (lower panel) three months after injury. There is a close spatial correlation between the T1-hyperintense signal and iron staining (arrowheads). **b** There is high spatial correlation between the area of T1 hyperintensities and iron deposits as assessed by Prussian blue staining. Pearson product-moment correlation analysis. **c** and **d** Iron deposits in pericontusional brain tissue assessed by longitudinal MRI. Extent of hemorrhage is comparable in RIPK1 (**c**) and RIPK3 (**d**) knockout animals and controls, indicating no differences in hemorrhage size after TBI between groups. T1 hyperintensities decreased over time in all groups, suggesting a very slow resorption of iron over time. **e.** Co-localization of iron deposits (red) observed at 1 month after TBI (upper panels) and lesion size assessed at the end of the observation period (3 months, middle row, green) suggests a progressive expansion of the lesion towards the regions with iron deposits. **f** and **g** Quantification of overlap between iron deposits and lesion. The higher the overlap of iron deposits and lesion size at three months, the higher the rate of tissue loss/ cell death in iron containing tissue. Co-localization is significantly less pronounced in RIPK1 (**f**) or RIPK3 (**g**) deficient mice, suggesting a reduced lesion growth in RIP knockouts due to toxic iron residues. Data are presented as mean ± SD; n = 9–10 for RIPK1, n = 8–9 for RIPK3. Two-way RM ANOVA with Tukey’s multiple comparisons test was used. **p < 0.005, ***p < 0.001

Since our data suggest that necroptotic signaling is important for chronic brain damage after TBI, we were interested to identify the mechanisms triggering this process. Traumatic contusions are associated with hemorrhage and subsequent deposition of iron in perilesional brain parenchyma. Since free iron is well-known to trigger ferroptotic [45] and possibly necroptotic cell death signaling [46], we hypothesized that chronic posttraumatic brain damage may be associated with iron deposition. Since iron can alter MRI signals, we looked for signal alterations using MRI scans. Indeed, we found hyperintense signals at the border of the lesion one month after TBI by T1-weighted MRI (Fig. 7a, upper panel) and could demonstrate that these signals showed a close spatial correlation with iron deposits as identified by Prussian blue staining (Fig. 7a, lower panel and Fig. 7b). Comparison of the volume of iron deposits between wild type and RIP deficient mice revealed equal amounts of iron in all animals, suggesting that the amount of hemorrhage was equal in all experimental groups (Fig. 7c and d). In a next step, we investigated the spatial and temporal relationship between chronic lesion expansion and iron deposits. For this purpose, we recorded iron deposits one month and lesion area three months after TBI, a time point when the lesion already expanded. In wild type mice the rim of the lesion area, the site of lesion progression, colocalized with iron deposits, while in neuronal specific RIPK1 deficient mice colocalization was minimal (Fig. 7e). The quantification of lesion area and iron deposition in wild type, RIPK1 and RIPK3 deficient mice, demonstrated that tissue loss colocalizing with histopathological detection of iron was significantly reduced in RIP deficient animals, suggesting that free iron may be involved in the pathophysiology of chronic neuronal necroptosis following TBI (Fig. 7f and g).

## Discussion

It is increasingly recognized that next to its acute sequelae, traumatic brain injury is a chronic disease [14]. Chronic post-trauma brain damage is associated with inflammation, persists for years after the initial insult, and may spread to areas initially not affected by the initial impact [47]. Affected patients often suffer from neuro-cognitive and mood disorders, personality changes, neurocognitive dysfunction, or even dementia [48–54]. So far, no therapeutic concepts targeting the long-term sequelae of TBI exist as the pathophysiology of chronic traumatic brain injury is still poorly understood.

Here, we propose, to our knowledge for the first time, that programmed cell death signaling mediates chronic neuronal injury after TBI. More specifically, we identified the necroptosis signaling molecules RIPK1 and RIPK3 to be major players in this process. Neurons affected by chronic traumatic damage showed necroptotic signaling as evidenced by enhanced levels of pMLKL. Moreover, *Ripk3* global knockout animals as well as neuronal RIPK1 deficient mice were significantly protected from chronic brain injury and showed improved neurocognitive function up to three months after TBI. Of note, no protection was observed within the first days after TBI, suggesting that necroptosis is not involved in acute injury, but specifically mediates chronic traumatic brain damage. Hence, our data further suggest that mechanistically acute and chronic neuronal cell death seem to be mediated by different processes.

Longitudinal MRI and histological assessment revealed that lesion progression was associated with parenchymal iron deposition and that neuronal or global deletion of RIPK1 or RIPK3 prevented lesion progression. These findings suggest that chronic traumatic brain damage may be triggered by iron and mediated by necroptotic signaling. The association of lesion progression with iron deposits is intriguing and may indicate that iron plays an important role in this process, however, further experiments addressing this issue in more detail will need to further evaluate whether there is a causal or just a correlative relationship between iron deposition and neuronal necroptosis.

Hemorrhage and the subsequent degradation of red blood cells releases large amounts of hemoglobin, heme, and free iron, i.e. molecules with high cytotoxic activity, into brain tissue [55]. From numerous studies investigating intraparenchymal hemorrhage, a subtype of hemorrhagic stroke, it is well known that specifically free iron generates reactive oxygen species thereby damaging cell membranes and causing tissue damage and neurological dysfunction [36, 56–59]. Cerebral macro- and microhemorrhages are common after TBI [60–62]. Specifically, microbleeds have been shown to exert toxic effects on endothelial cells, astrocytes, neurons, oligodendrocytes, and microglia and may thus lead to blood–brain barrier damage, neuronal cell death, demyelination, and chronic inflammation [63]. The importance of blood degradation products for the pathophysiology of TBI is further demonstrated by the fact that the presence and extent of hemorrhage show a close correlation with injury severity and long-term clinical outcome in TBI patients [63, 64]. In line with these clinical studies, we previously demonstrated that the TBI model used in current study shows acute macro-hemorrhage, which, however, resolves within the first week after injury [23]. In the current study we now show by MRI and Prussian blue staining that iron persists in pericontusional tissue for up to three months after the initial impact. Iron deposits identified in still viable pericontusional brain tissue one month after TBI co-localized with damaged tissue three months after injury, suggesting that chronic lesion progression preferably occurred in areas with previous hemorrhage and subsequent iron deposition. Since this process was significantly attenuated in neuronal *Ripk1* and global *Ripk3* knockout animals, our findings suggest that pericontusional iron may be involved in chronic posttraumatic lesion expansion and that this process is mediated by necroptotic signaling in neurons.

So far, neuroinflammation was believed to be the main cause of chronic brain damage after TBI [44]. However, the mechanisms by which microglial activation promotes neuronal injury and death remain elusive. Further, microglial activation after TBI may exert beneficial as well as detrimental effects, i.e. tissue regeneration versus accelerated damage, respectively. Therefore, disentangling these opposite functions of microglia may have important therapeutic consequences. Our current data suggest that the final steps causing neuronal cell death during chronic post-trauma brain damage depend on RIPK1 and RIPK3 activity. Based on these findings, we suggest a hypothetical scenario in which chronic post-trauma brain damage is initiated by the ongoing production of reactive oxygen species (ROS) by inflammatory cells. Physiological concentrations of ROS are usually well tolerated by cells since they are detoxified to water and oxygen by the glutathione system and catalases [65]. However, in the presence of iron, hydrogen peroxide is converted to highly reactive hydroxyl radicals by the Fenton reaction and may initiate a form of programmed cell death called ferroptosis [66, 67]. The link between ferroptosis and RIPK1/3-mediated necroptosis in neurons is not fully established, but may be mediated by cylindromatosis (Cyld), a deubiquitinase able to activate RIPK1 and downstream necroptosome formation under conditions of oxidative stress as we recently demonstrated [21]. We showed that CYLD-dependent RIPK1/RIPK3 necrosome-formation occurred in neuronal cells exposed to ferroptosis activators and knockdown of the necroptosis-mediators CYLD, RIPK1 or RIPK3 attenuated cell death. Interestingly, the role for CYLD in ferroptosis also translated into neuroprotective effects in vivo, since CYLD knockout mice showed reduced secondary brain damage after TBI compared to controls [21]. These findings are corroborated by results in models of hemorrhagic stroke suggesting that necroptosis and ferroptosis are indeed interconnected under conditions of blood-induced tissue damage [68].

Despite its obvious strengths, the current study also has some notable limitations. We studied only young male animals and are therefore not able to make any statements on the role of necroptosis in the aged or female brain. Further, due to technical limitations, such as the lack of specific antibodies for the study of necroptosis in brain tissue, we were only able to demonstrate the involvement of a single signaling molecule downstream of RIPK activation, namely pMLKL. Thus, future studies using novel experimental tools will need to define necroptotic signaling after TBI in more detail. Another shortcoming of the current study is that we demonstrate only a spatial correlation between iron deposition and necroptosis. Thus, further studies are needed to further clarify whether there is a causal relationship between iron deposition and neuronal necroptosis. Finally, we want to point out that memory tests in mice are sometimes hard to interpret since results may be influenced by differences in motor function or the level of disinhibition which are well known to occur after TBI. We controlled for differences in motor function and the intensity of exploratory behavior between groups and are confident that the presented data indeed reflect memory function, however, the results need nevertheless to be interpreted with caution.

In conclusion, the current study provides evidence that RIPK1 and RIPK3 are critically involved in chronic post-trauma brain damage. Further, our findings suggest that free iron may be involved in this process. Our results therefore help to better understand the mechanisms of chronic post-trauma brain damage and suggest that RIPK1- and RIPK3-mediated necroptosis may represent a novel therapeutic target for the treatment of patients suffering from the long-term sequels of TBI.

## Supplementary Information

**Additional File 1 Supplementary Fig. S1.** Genotyping of RIPK1 and RIPK3 deficient mice and proof of neuronal specific RIPK1 knock-out in RIPK1^flox/flox^Camk2CreERT2 mice. **a.** Neuron specific RIPK1 deficient mice used for experiments were heterozygous for Camk2CreERT2 and homozygous for the floxed RIPK1 allele. Littermate controls were also homozygous for the floxed RIPK1 allele, but did not express the Cre recombinase. **b.** Global RIPK3 deficient mice were homozygous for disrupted allele, while control mice expressed only the wild type gene. **c. and d.** To demonstrate specific neuron specific RIPK1 deficiency in induced RIPK1^flox/flox^Camk2CreERT2 mice, we performed immunohistochemistry for RIPK1 and NeuN, a neuronal marker. In the cortex of control mice RIPK1 was almost exclusively expressed in neurons (upper panels), while in induced RIPK1^flox/flox^Camk2CreERT2 mice RIPK1 staining was significantly reduced (lower panels) to 20% of baseline (d).
**Additional File 1 Supplementary Fig. 2** Body weight and physical condition after experimental TBI. **a. and b.** Weight after TBI. Animals recovered from weight loss directly after trauma within one week after injury; in the following observation period weight constantly increased. No differences were detected between CCI and sham-operated animals in the RIPK1 (**a**) and RIPK3 (**b**) groups. **c. and d.** General health score to assess recovery. All animals’ general condition transiently worsened in the perioperative phase with a peak at day 1 after TBI, but returned to baseline within one week. There was no difference between groups, **c.** RIPK1, **d.** RIPK3. Data are presented as mean ± SD; n = 5 for sham, n = 8–10 for TBI.
**Additional File 1 Supplementary Fig. 3**. Individual lesion volume progression for a. RIP1 and b. RIP3 deficient mice.
**Additional File 1 Supplementary Fig. 4**. RIPK3 deficiency does not affect acute brain injury after TBI. No differences in lesion volume as assessed by histology was detected between RIPK3 knockout mice and C57BL/6 wild type controls at 24 h after TBI. Data are presented as mean ± SD; n = 10.
**Additional File 1 Supplementary Fig. 5**. Lesion volumes by MRI and histology. **a.** T2-weighted MRI and Nissl stained coronal section three months after injury. Lesion volume was quantified in T2-weighted MRI images as well as Nissl stained sections obtained in the same animals three months post injury. **b.** Correlation analysis of lesion volumes assessed by histomorphometry or T2-weighted MRI revealed a strong positive correlation between both methods. Differences in measured lesion size are due to shrinking of tissue in the fixation process, with a shrinking factor of 0.77 ± 0.23. **c.—f.** Quantification of lesion volume (**c.** RIPK1, **d.** RIPK3) as well as hippocampal damage (**e.** RIPK1, **f.** RIPK3) using both methods. Data are presented as mean ± SD; n = 8–10 for TBI. Pearson correlation, Students t-test for parametric and Man-Whitney-Rank-Sum test for non-parametric data were used. *p < 0.05, ***p < 0,001.

## Data Availability

The datasets used and/or analyzed during the current study available from the corresponding author on reasonable request.
