# Supplementary material for: RIPK1 or RIPK3 deletion prevents progressive neuronal cell death and improves memory function after traumatic brain injury
Source: Acta Neuropathol Commun. 2021 Aug 17;9:138. doi: 10.1186/s40478-021-01236-0 (PMC8369637; doi:10.1186/s40478-021-01236-0)
Supplement: Supplementary file 1 — Additional File 1 Supplementary Fig. S1. Genotyping of RIPK1 and RIPK3 deficient mice and proof of neuronal specific RIPK1 knock-out in RIPK1flox/floxCamk2CreERT2 mice. a. Neuron specific RIPK1 deficient mice used for experiments were heterozygous for Camk2CreERT2 and homozygous for the floxed RIPK1 allele. Littermate controls were also homozygous for the floxed RIPK1 allele, but did not express the Cre recombinase. b. Global RIPK3 deficient mice were homozygous for disrupted allele, while control mice expressed only the wild type gene. c. and d. To demonstrate specific neuron specific RIPK1 deficiency in induced RIPK1flox/floxCamk2CreERT2 mice, we performed immunohistochemistry for RIPK1 and NeuN, a neuronal marker. In the cortex of control mice RIPK1 was almost exclusively expressed in neurons (upper panels), while in induced RIPK1flox/floxCamk2CreERT2 mice RIPK1 staining was significantly reduced (lower panels) to 20% of baseline (d). Additional File 1 Supplementary Fig. 2 Body weight and physical condition after experimental TBI. a. and b. Weight after TBI. Animals recovered from weight loss directly after trauma within one week after injury; in the following observation period weight constantly increased. No differences were detected between CCI and sham-operated animals in the RIPK1 (a) and RIPK3 (b) groups. c. and d. General health score to assess recovery. All animals’ general condition transiently worsened in the perioperative phase with a peak at day 1 after TBI, but returned to baseline within one week. There was no difference between groups, c. RIPK1, d. RIPK3. Data are presented as mean ± SD; n = 5 for sham, n = 8–10 for TBI. Additional File 1 Supplementary Fig. 3. Individual lesion volume progression for a. RIP1 and b. RIP3 deficient mice. Additional File 1 Supplementary Fig. 4. RIPK3 deficiency does not affect acute brain injury after TBI. No differences in lesion volume as assessed by histology was detected between RIPK3 knockout mice and C57BL/6 wil [file 40478_2021_1236_MOESM1_ESM.pptx]

## Slide 1
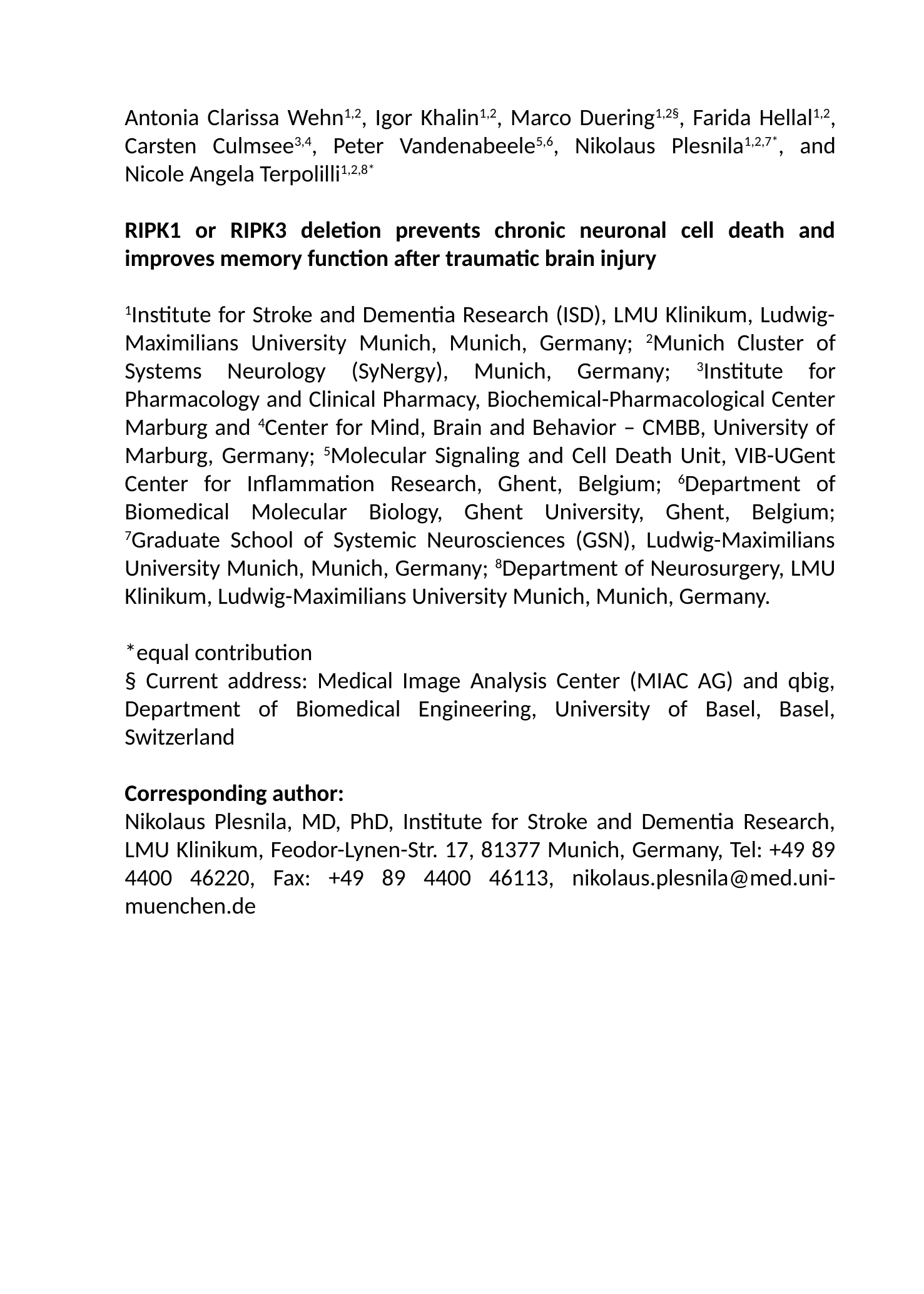

Antonia Clarissa Wehn1,2, Igor Khalin1,2, Marco Duering1,2§, Farida Hellal1,2, Carsten Culmsee3,4, Peter Vandenabeele5,6, Nikolaus Plesnila1,2,7*, and Nicole Angela Terpolilli1,2,8*
RIPK1 or RIPK3 deletion prevents chronic neuronal cell death and improves memory function after traumatic brain injury
1Institute for Stroke and Dementia Research (ISD), LMU Klinikum, Ludwig-Maximilians University Munich, Munich, Germany; 2Munich Cluster of Systems Neurology (SyNergy), Munich, Germany; 3Institute for Pharmacology and Clinical Pharmacy, Biochemical-Pharmacological Center Marburg and 4Center for Mind, Brain and Behavior – CMBB, University of Marburg, Germany; 5Molecular Signaling and Cell Death Unit, VIB-UGent Center for Inflammation Research, Ghent, Belgium; 6Department of Biomedical Molecular Biology, Ghent University, Ghent, Belgium; 7Graduate School of Systemic Neurosciences (GSN), Ludwig-Maximilians University Munich, Munich, Germany; 8Department of Neurosurgery, LMU Klinikum, Ludwig-Maximilians University Munich, Munich, Germany.
*equal contribution
§ Current address: Medical Image Analysis Center (MIAC AG) and qbig, Department of Biomedical Engineering, University of Basel, Basel, Switzerland
Corresponding author:
Nikolaus Plesnila, MD, PhD, Institute for Stroke and Dementia Research, LMU Klinikum, Feodor-Lynen-Str. 17, 81377 Munich, Germany, Tel: +49 89 4400 46220, Fax: +49 89 4400 46113, nikolaus.plesnila@med.uni-muenchen.de

## Slide 2
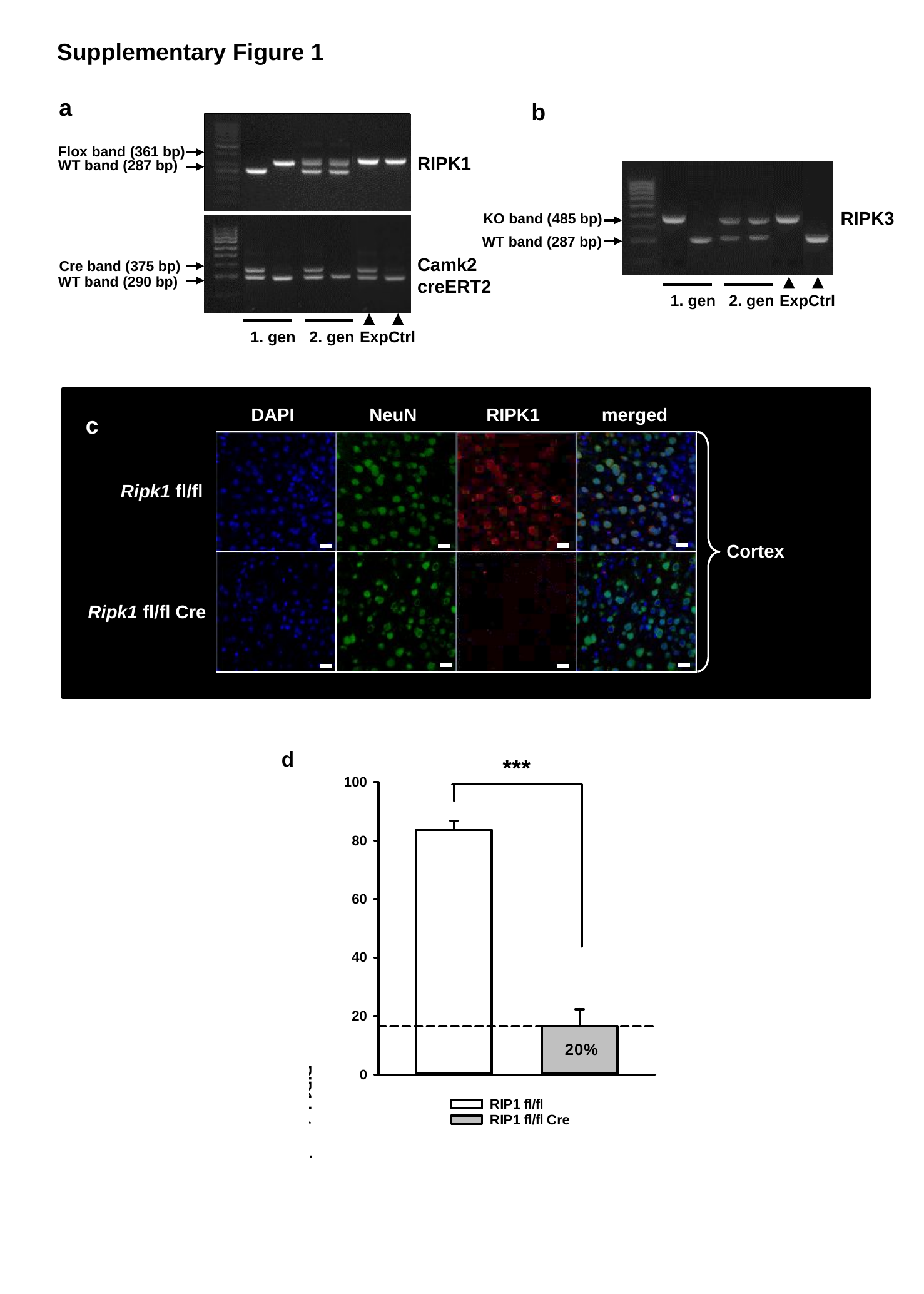

Supplementary Figure 1
a
Flox band (361 bp)
RIPK1
WT band (287 bp)
Camk2
creERT2
Cre band (375 bp)
WT band (290 bp)
1. gen
2. gen
Exp
Ctrl
b
RIPK3
KO band (485 bp)
WT band (287 bp)
1. gen
2. gen
Exp
Ctrl
DP
DAPI
NeuN
RIPK1
merged
c
Ripk1 fl/fl
Cortex
Ripk1 fl/fl Cre
d

## Slide 3
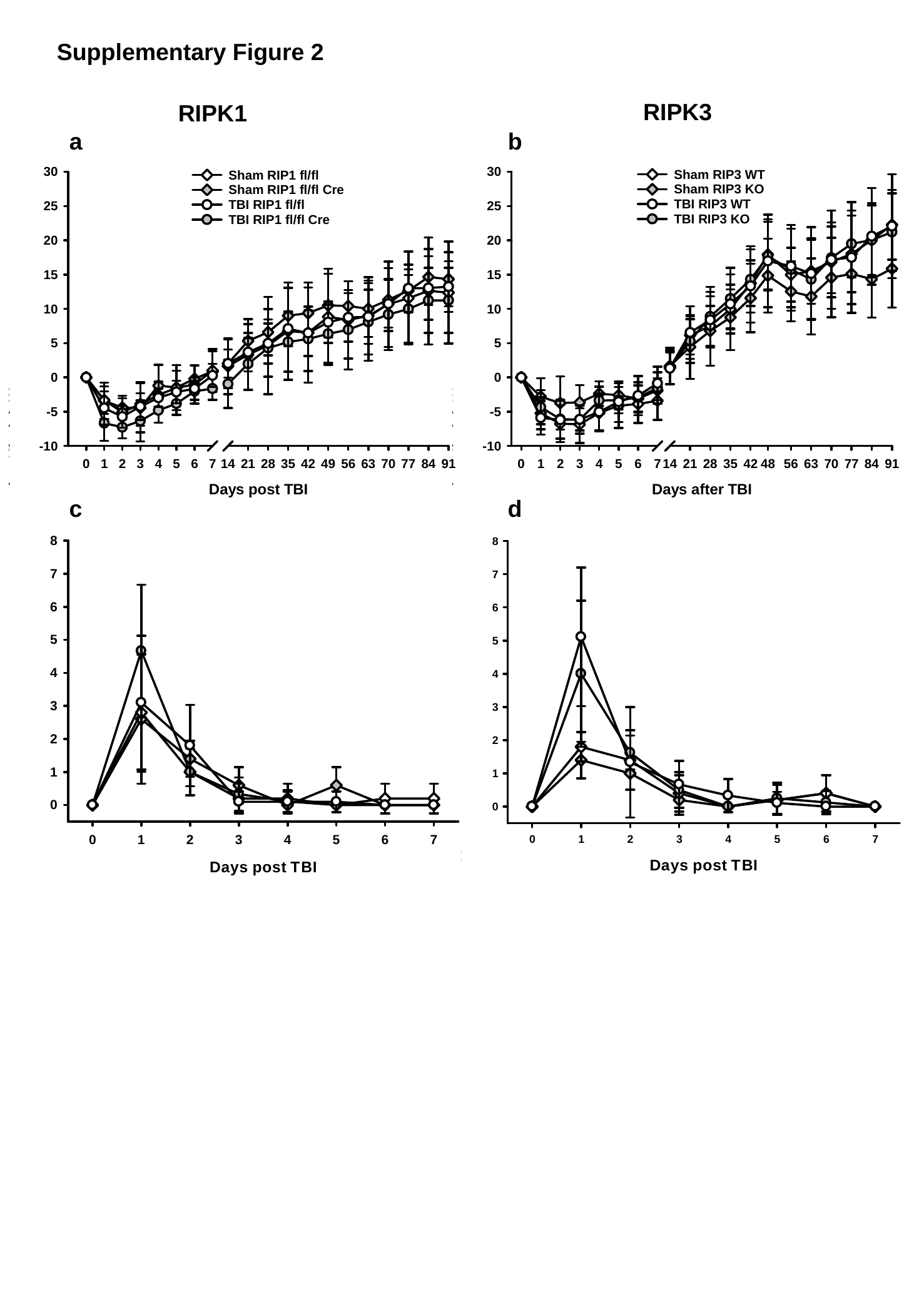

Supplementary Figure 2
RIPK3
RIPK1
a
b
c
d

## Slide 4
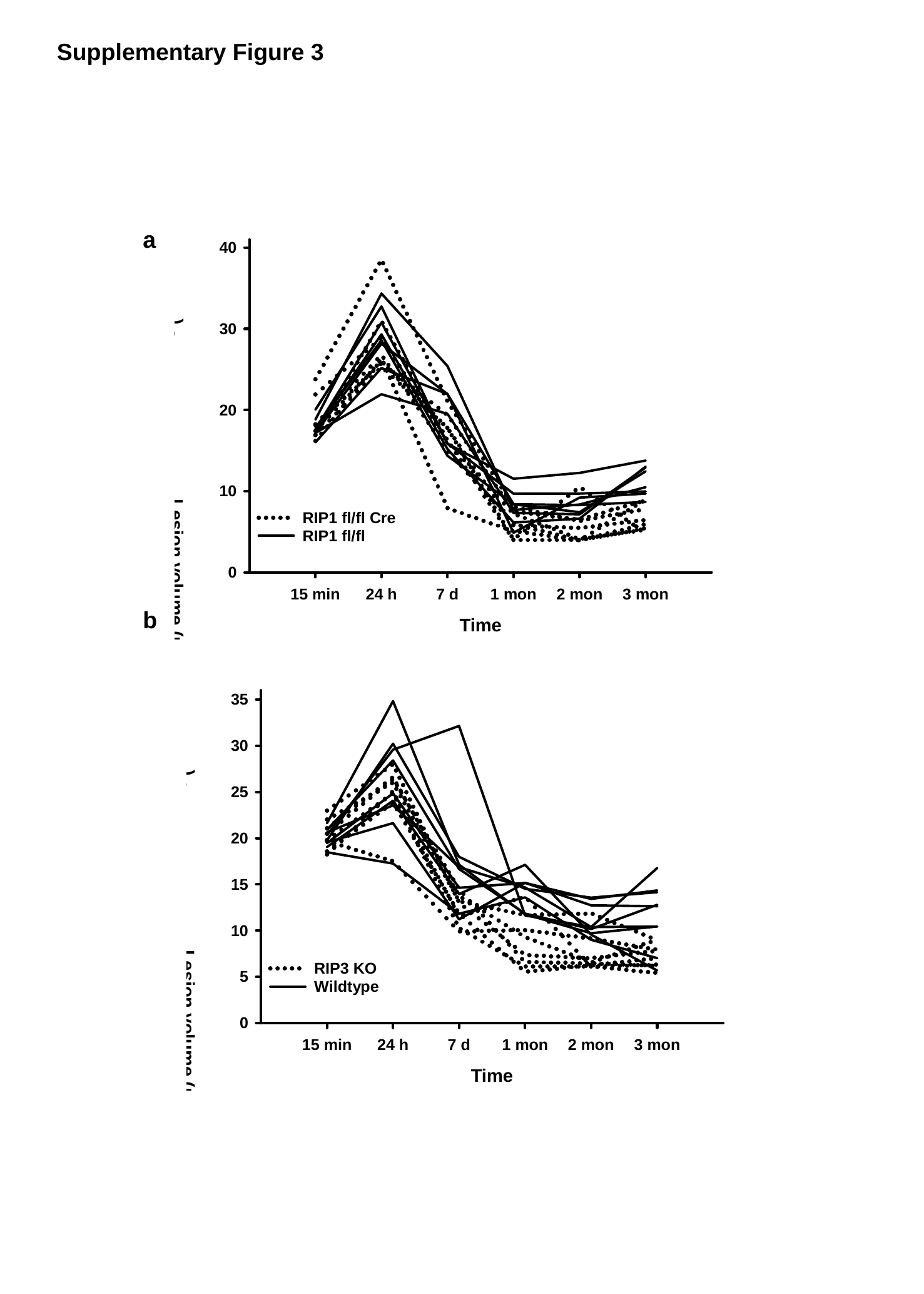

Supplementary Figure 3
a
b

## Slide 5
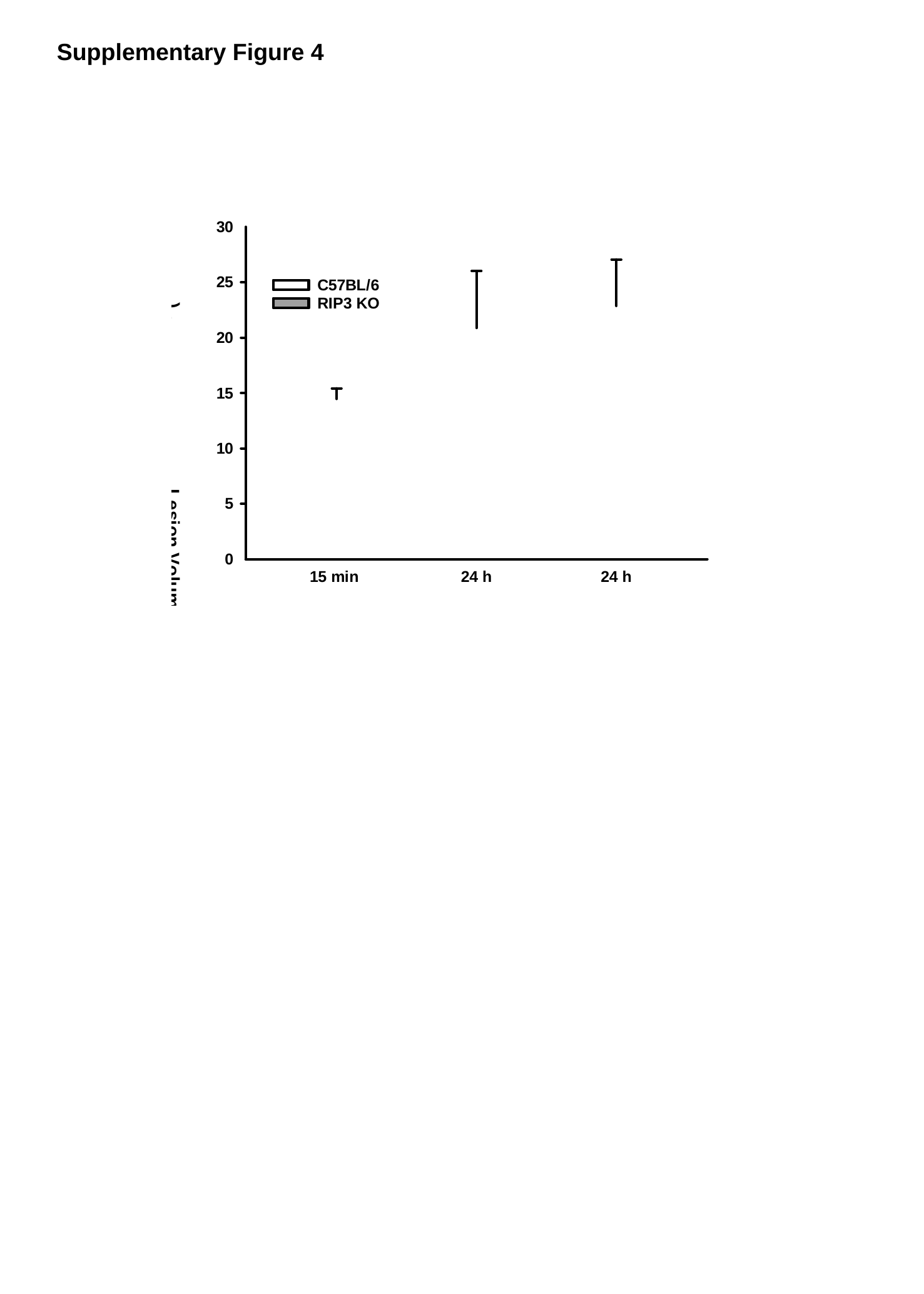

Supplementary Figure 4

## Slide 6
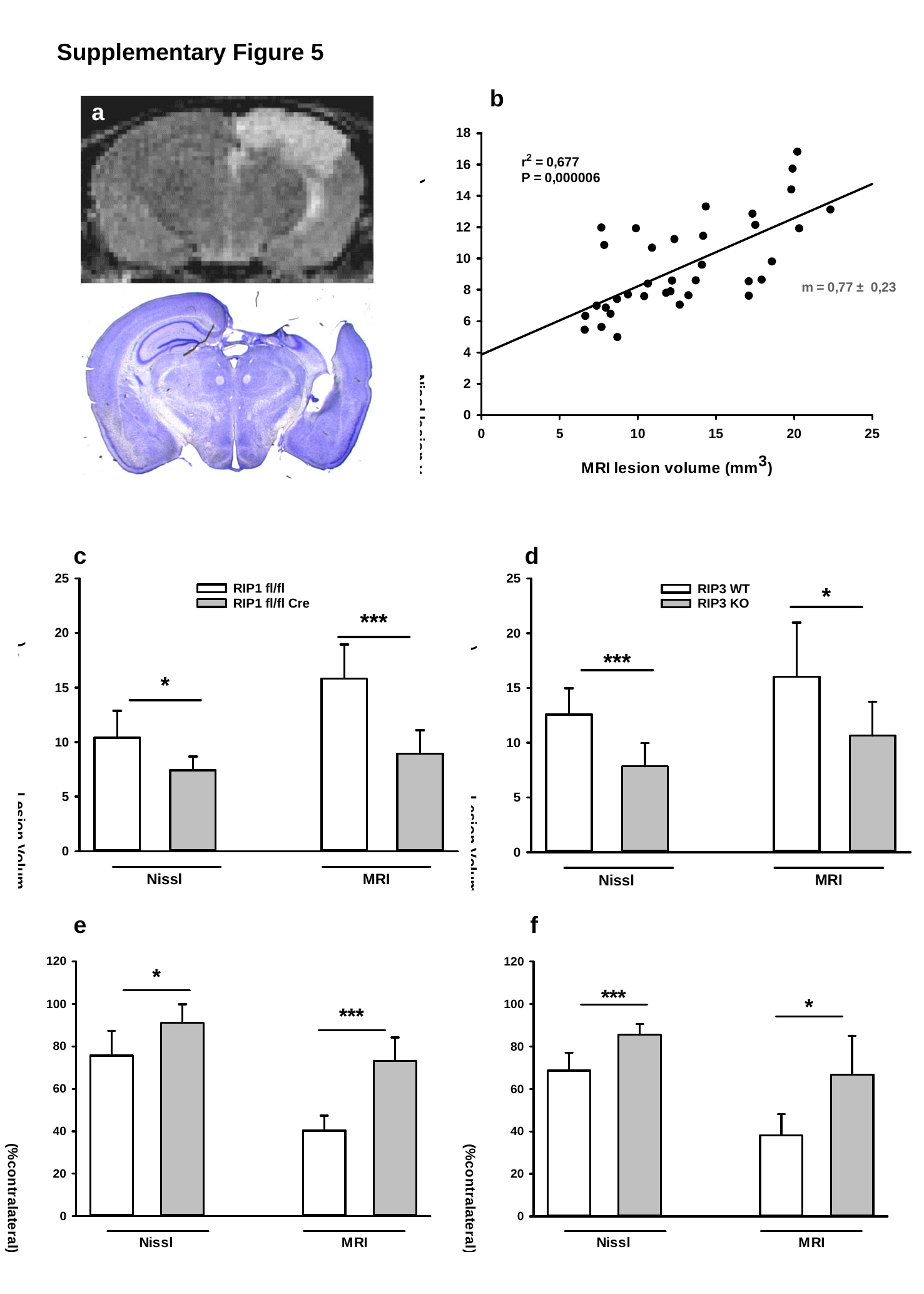

Supplementary Figure 5
b
a
c
d
e
f
